# Association between Metformin and a Lower Risk of Age-Related Macular Degeneration in Patients with Type 2 Diabetes

**DOI:** 10.1155/2019/1649156

**Published:** 2019-10-31

**Authors:** Yu-Yen Chen, Ying-Cheng Shen, Yun-Ju Lai, Chun-Yuan Wang, Keng-Hung Lin, Shih-Chao Feng, Chiao-Ying Liang, Li-Chen Wei, Pesus Chou

**Affiliations:** ^1^Department of Ophthalmology, Taichung Veterans General Hospital, Taichung 407, Taiwan; ^2^School of Medicine, National Yang-Ming University, Taipei 112, Taiwan; ^3^Community Medicine Research Center and Institute of Public Health, National Yang-Ming University, Taipei 112, Taiwan; ^4^Division of Endocrinology and Metabolism, Department of Internal Medicine, Puli Branch of Taichung Veterans General Hospital, Nantou 545, Taiwan; ^5^Department of Exercise Health Science, National Taiwan University of Sport, Taichung 404, Taiwan

## Abstract

**Purpose:**

This population-based, retrospective cohort study was to investigate whether metformin is associated with a lower risk of subsequent age-related macular degeneration (AMD) in patients with type 2 diabetes.

**Methods:**

Using the Taiwan National Health Insurance Research Database from 2001 to 2013, 68205 subjects with type 2 diabetes were enrolled in the study cohort. Among them, 45524 were metformin users and 22681 were nonusers. The metformin and nonmetformin groups were followed until the end of 2013. Cox regression analyses were used to estimate hazard ratios (HRs) for AMD development associated with metformin use. Confounders included for adjustment were age, sex, and comorbidities (hypertension, hyperlipidemia, coronary artery disease, obesity, diabetic retinopathy, chronic kidney disease, and insulin treatment). Furthermore, propensity score (PS) matching method was used to choose the matched sample, and PS-adjusted Cox regression was performed. Finally, how HRs changed according to metformin treatment duration and dose was also evaluated in the metformin group.

**Results:**

After adjusting for confounders, the metformin group had a significantly lower risk of AMD (adjusted HR = 0.54; 95% confidence interval [CI], 0.50–0.58). In the PS-matched sample, the significance remained (adjusted HR = 0.57; 95% CI, 0.52–0.63). In the metformin group, the adjusted HRs for the second (1.5–4 years) and third (≥4 years) tertiles of metformin treatment duration were 0.52 and 0.14, respectively, compared with the first tertile (<1.5 years). We also found significant trends of lower HRs (all *p*-value for trend <0.05) with increasing total and average doses.

**Conclusions:**

Among patients with type 2 diabetes, those who use metformin are at a significantly lower risk of developing AMD relative to individuals who do not use metformin. Also, the trend of a significantly lower AMD risk was found with a higher dose of metformin.

## 1. Introduction

Metformin, classified as biguanide, is the first-line and most widely used medication for patients with type 2 diabetes. In addition to its main effect of inhibiting hepatic glucose production [1], metformin has been found to decrease oxidative stress [2, 3]. Furthermore, recent clinical studies have suggested that metformin suppresses inflammation through direct and indirect pathways [3, 4].

Age-related macular degeneration (AMD) is the leading cause of irreversible blindness in people older than 50 years in developed countries [5, 6]. The number of patients with AMD is estimated to reach 196 million by 2020 and 288 million by 2040, based on population-aging demographics [7]. The deposition of drusen in the retina is the earliest sign of AMD. Advanced AMD is characterized by progressive retinal atrophy or choroidal neovascularization. Its pathogenesis is not fully understood but has been found to be related to oxidative stress, inflammation, and immune reaction [8–10]. As metformin has been found to have antioxidative and anti-inflammatory properties, it is relevant to propose that metformin may be associated with the lower occurrence of AMD. Unlike diabetic retinopathy that is a classical complication of diabetes, AMD is not directly induced by hyperglycemia. Therefore, the possible association between metformin and a lower AMD risk may not be related to the glucose-lowering effect of metformin.

Previous studies have shown that metformin can reduce the incidence of cardiovascular events and all-cause mortality [11–14]. Evans et al. [15] reported a significant reduction in the incidence of malignant cancer with metformin use. In addition, metformin was observed to retard aging and extend the lifespan in animals [16, 17]. However, to the best of our knowledge, no study has investigated the association between metformin use and the lower risk of AMD in patients with type 2 diabetes, perhaps due to the difficulty of undertaking a long-term follow-up study with sufficient case numbers, correct diagnoses, and detailed prescription records. Besides, in such a study, risk factors for AMD, such as age and comorbidities (e.g., hypertension, hyperlipidemia, coronary artery disease, obesity, diabetic retinopathy, and chronic kidney disease) should be adjusted for as confounders to clarify the relationship between metformin and AMD. Taiwan's National Health Insurance Research Database (NHIRD) includes comprehensive diagnoses from 1996 to 2013, which are registered using the International Classification of Diseases, Ninth Revision, Clinical Modification (ICD-9-CM) codes and have been verified using standard diagnostic criteria. Furthermore, the NHIRD includes exhaustive prescription registries. Therefore, research on the relationship between metformin use and AMD can be conducted using the database.

The study hypothesis was that metformin use would reduce the risk of subsequent AMD development in patients with type 2 diabetes. Utilizing NHIRD data, we investigated whether metformin was associated with a lower AMD risk after adjustment for potential confounders. We also examined whether the metformin treatment duration or dose affected the development of AMD.

## 2. Materials and Methods

### 2.1. Data Source

Taiwan's National Health Insurance program covers more than 99% of the country's 23 million residents. The NHIRD is maintained by the National Health Research Institutes of Taiwan and consists of inpatient and outpatient medical benefit claims, including diagnostic codes, prescriptions, operative procedures, and insurance registry data. The identification of all patients included in the database was encrypted prior to the release of data for research purposes. This study utilized the Longitudinal Health Insurance Database (LHID), which is a representative database containing data from 1000000 individuals sampled randomly from all beneficiaries in NHIRD. The Institutional Review Board waived the requirement for informed consent because the secondary data were scrambled and deidentified before release. This study was approved by the ethics committee of Yang-Ming University Hospital (2015A018).

### 2.2. Study Design and Sample

We conducted a retrospective cohort study for the period from 1 January 2001 to 31 December 2013. First, we selected patients from the LHID who were diagnosed with diabetes (ICD-9-CM code 250) during the study period. Those diagnosed with diabetes before the end of 2000 and those diagnosed with type 1 diabetes before the end of 2013 were excluded to ensure that all enrolled patients had new-onset type 2 diabetes. The date of first type 2 diabetes diagnosis was defined as the index date. Second, patients in the study cohort were followed to identify the onset of AMD (ICD-9-CM codes 362.50, 362.51, 362.52), which had to be confirmed by fundoscopy, fluorescein angiography, and/or optical coherence tomography. Those diagnosed with AMD before the index date were excluded to ensure that all included AMD cases were new onset after the diagnosis of diabetes. The study cohort was followed until the occurrence of AMD, dropout from the database, or the end of 2013, whichever came first.

### 2.3. Variable Definitions

As this study examined the impact of metformin on the risk of AMD, the main independent variable of interest was metformin use. Metformin was identified and classified by the National Drug Code and the Anatomic Therapeutic Chemical code. Previous studies revealed that age, gender, and some specific comorbidities may be risk factors of AMD. These factors were confounders and had to be adjusted in our statistical analyses. We derived information regarding age and gender in the registration files of beneficiaries. The specific comorbidities were identified by the ICD-9-CM codes in the inpatient and ambulatory care expenditure files. These comorbidities included hypertension (ICD-9-CM codes 401–405), hyperlipidemia (ICD-9-CM code 272), coronary artery disease (ICD-9-CM codes 410–414), obesity (ICD-9-CM codes 278.0, 278.1), and diabetic retinopathy (ICD-9-CM codes 362.01–362.02). Laboratory data regarding the kidney function (e.g., estimated glomerular filtration rate; eGFR) or glucose control (e.g., hemoglobin A1c; HbA1c) were not available in our database. Therefore, we adopted the diagnosis of chronic kidney disease (ICD-9-CM code 585) as the surrogate of impaired kidney function and insulin treatment as a surrogate of poor glycemic control. The use of other antidiabetic medications, antihypertensives, and lipid-lowering drugs were also assessed from the prescription registries.

### 2.4. Statistical Analysis

First, characteristics of the study cohort were presented according to age, sex, hypertension, hyperlipidemia, coronary artery disease, obesity, diabetic retinopathy, chronic kidney disease, insulin treatment, and concomitant medications (other antidiabetic oral medications, antihypertensives, and lipid-lowering agents). Individuals were classified as metformin users (metformin group) if they ever used metformin during the follow-up period. Nonusers (nonmetformin group) were those who never used metformin. Then, differences in covariate characteristics between groups were assessed using the two-sample *t*-test (for continuous variables) and the chi-square test (for categorical variables). Survival analyses using the Kaplan–Meier curves and the log-rank test were performed to compare the cumulative hazards for AMD between the two groups. Second, a Cox proportional hazard model was used to estimate unadjusted hazard ratios (HRs) for AMD according to each variable in univariate analyses. Adjusted HRs for AMD were then derived from multivariate Cox regression analyses. Variables included in the multivariate analyses were age, sex, metformin use, comorbidities (hypertension, hyperlipidemia, coronary artery disease, obesity, and diabetic retinopathy), as well as chronic kidney disease and insulin treatment. Metformin use, comorbidities, chronic kidney disease, and insulin treatment were regarded as time-dependent variables.

Additionally, we conducted analysis with the propensity score (PS) matching method. PS of each individual was computed from all the confounders included in the previous Cox regression. Also adopted to generate PS were confounders such as antidiabetic oral drugs (other than metformin), antihypertensives, and lipid-lowering agents. The nonmetformin group and the metformin group were chosen to be one to one matched on PS, thus reducing the confounding effects. Thereafter, we applied the PS-adjusted Cox regression to derive the impact of metformin on AMD risk.

Among metformin users, we then investigated whether the lower risk of AMD was dose responsive to metformin. The treatment duration, total dose, and average dose of metformin were calculated from the prescription records. Each of the three items was categorized into tertiles. Adjusted HRs for the second and third tertiles of each item were compared to the corresponding first tertile, and *p*-values for trends were derived to verify the dose-response relationship between metformin use and the lower risk of AMD. All statistical operations were performed using SAS statistical package, version 9.2 (SAS Institute, Cary, NC, USA).

## 3. Results

### 3.1. Demographic and Clinical Characteristics of the Study Sample

**Figure 1:**
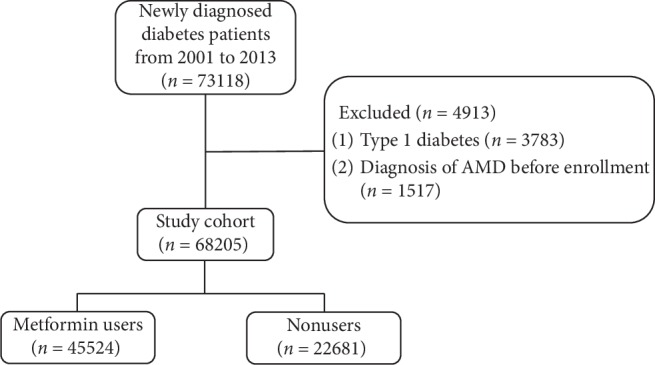
Flow chart showing the procedures in selecting the participants into the study. AMD: age-related macular degeneration.

Figure 1 shows the flowchart for the enrollment of study participants. In total, 73178 patients were newly diagnosed with diabetes from 2001 to 2013. After the exclusion of patients with type 1 diabetes (*n* = 3783) and those diagnosed with AMD prior to the diagnosis of diabetes (*n* = 1517), 68205 patients with type 2 diabetes were included in the study cohort. Of them, 45524 were metformin users and 22681 were nonusers.  Table 1 presents the demographic and clinical characteristics of the metformin and nonmetformin groups. The mean age in the overall study cohort was 56.1 years. People in the metformin group were significantly younger and had higher prevalences of hypertension, hyperlipidemia, coronary artery disease, obesity, and diabetic retinopathy; in addition, males were more predominant in this group. The prevalences of chronic kidney disease, the proportion of insulin treatment, and the distributions of concomitant medications (other antidiabetic oral medications, antihypertensives, and lipid-lowering agents) revealed significant differences between the two groups. During the study period, the frequency of AMD was significantly lower in the metformin group (3.4%) than the nonmetformin group (5.6%).

### 3.2. Kaplan–Meier Curves and Log-Rank Test

**Figure 2:**
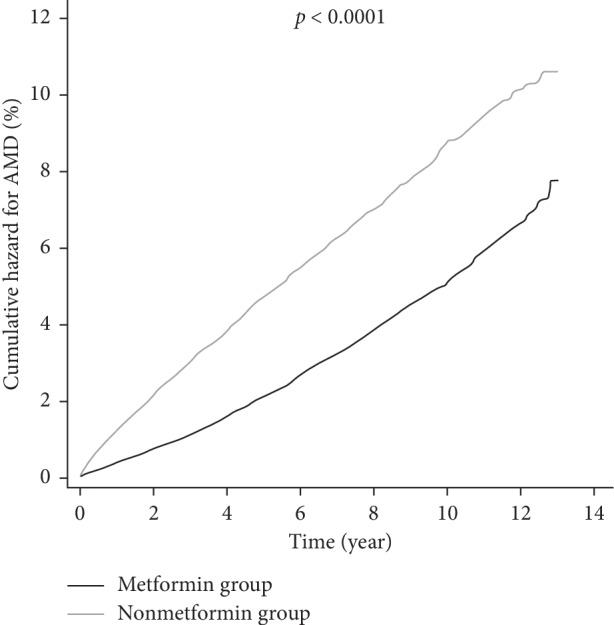
Kaplan–Meier curves for AMD among the metformin group and the nonmetformin group. The black line represents the metformin group and the gray line represents the nonmetformin group. AMD: age-related macular degeneration.

Figure 2 illustrates the cumulative hazards for AMD in the metformin group and the nonmetformin group. The log-rank test revealed that the metformin group had a significantly lower cumulative hazard for AMD than the nonmetformin group (*p* < 0.0001).

### 3.3. AMD Risk

Table 2 displays HRs for AMD with regard to age, sex, metformin use, comorbidities, chronic kidney disease, and insulin treatment. The univariate Cox regression analysis yielded an unadjusted HR for AMD of 0.52 (95% confidence interval [CI], 0.48–0.57) for the metformin group compared with the nonmetformin group. After adjusting for covariates, the significantly lower HR for AMD in the metformin group remained (adjusted HR = 0.54; 95% CI, 0.50–0.58). Age was a significant risk factor for AMD in univariate and multivariate analyses. The adjusted HR for AMD in patients over 70 years of age compared with those younger than 50 years was 6.44. In the univariate and multivariate analyses, patients with comorbidities such as hypertension, hyperlipidemia, coronary artery disease, and obesity had a significantly higher risk of developing AMD. Diabetic retinopathy also significantly increased the risk for AMD in the univariate analysis (unadjusted HR = 1.51; 95% CI, 1.37–1.67) and in the multivariate analysis (adjusted HR = 1.98; 95% CI, 1.78–2.20). Chronic kidney disease was a significant risk factor for AMD in the univariate analyses; however, the significance did not remain after adjustment of confounders. Besides, patients with insulin treatment did not have a significantly higher risk of AMD in the univariate and multivariate analyses.

### 3.4. HRs for AMD Calculated by PS-Matching Method

Table 3 shows the characteristics of the PS-matched sample. The metformin group and the nonmetformin group were one to one matched on the PS. Compared with the original sample, the PS-matched sample had more similar distributions of confounders in the two groups. Besides, PS in the two groups revealed no significant difference (*p*=0.80), supporting that the two groups were well-matched.

In Table 4, we computed the HR for AMD using the PS-adjusted Cox regression. After adjustment of PS in the Cox regression, metformin users still had a significantly lower risk of developing AMD (adjusted HR = 0.57; 95% CI, 0.52–0.63).

AMD: age-related macular degeneration; PS: propensity score; HR: hazard ratio; CI: confidence interval. Metformin use and PS are included in the regression analysis.

### 3.5. Effects of the Metformin Treatment Duration and Dose on AMD Risk

**Figure 3:**
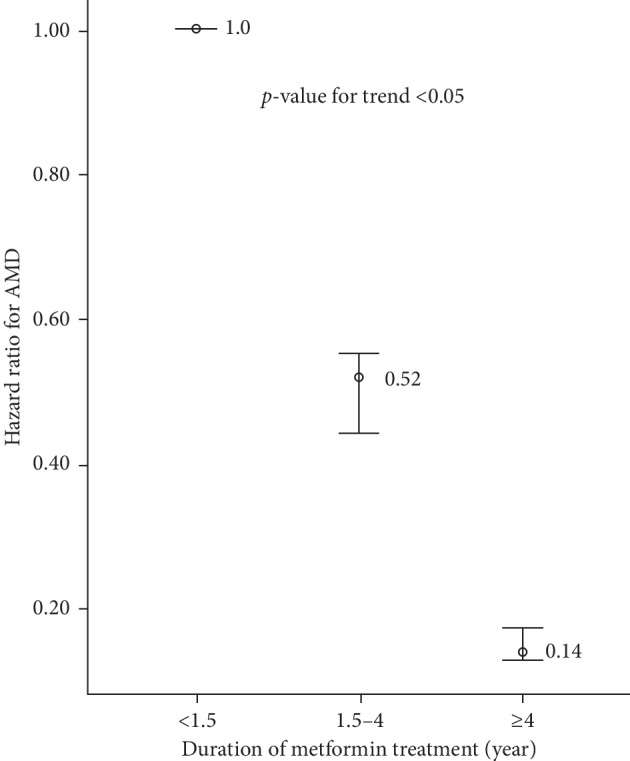
Risk of AMD according to metformin treatment duration. AMD: age-related macular degeneration.

Figure 3 illustrates changes in the adjusted HR for AMD with the duration of metformin use. Adjusted HRs obtained by comparing the second (1.5–4 years) and third (≥4 years) tertiles to the first tertile (<1.5 years) were 0.52 (95% CI, 0.44–0.55) and 0.14 (95% CI, 0.13–0.17), respectively. Trend analysis revealed a significant change (*p*-value for trend <0.05). Thus, a longer metformin treatment duration was associated with a significant tendency for a lower AMD risk.

**Figure 4:**
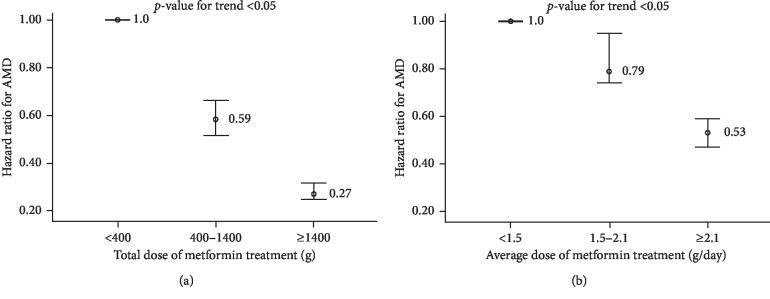
Risk of AMD according to (a) total dose and (b) average dose of metformin. AMD: age-related macular degeneration.

Figure 4 displays the risk of AMD corresponding to the dose of metformin. The total dose (Figure 4(a)) and the average dose (Figure 4(b)) were included separately in Cox regression analyses. Regarding the total dose of metformin, adjusted HRs for AMD in the second (400–1000 g) and third (≥1000 g) tertiles compared with the first tertile were 0.59 (95% CI, 0.52–0.66) and 0.27 (95% CI, 0.25–0.32), respectively (*p*-value for trend <0.05). We also found a trend of significantly lower AMD risk with increasing average metformin dose (adjusted HRs = 0.79 [second tertile] and 0.53 [third tertile]; *p*-value for trend <0.05).

## 4. Discussion

This study is the first to reveal the association between metformin and a lower risk of AMD development. In our population-based study utilizing Taiwan's NHIRD with a long (13-year) study period, we found that in patients with type 2 diabetes, the risk of AMD was significantly lower among those who used metformin than those who did not. Even after adjustment for confounders in the original sample or in the PS-matched sample, the results remained significant. We also detected trends of lower risk of AMD development with longer treatment duration and higher total and average doses of metformin. AMD is a multifactorial disease, and advanced age is a main risk factor. In our study, the metformin group was significantly younger than the nonmetformin group, which is compatible with previous studies [18–20]. The distribution of comorbidities also differed significantly between the metformin and nonmetformin groups. Thus, all such demographic and clinical variables should be adjusted for as confounders in evaluations of the risk of AMD development.

In previous studies, hypertension [21], hyperlipidemia [22], coronary artery disease [23], and obesity [24] have been found to be associated with AMD. In univariate and multivariate Cox regression analyses of our study, age and these comorbidities were also found to be significant risk factors for AMD. Notably, our study revealed that diabetic retinopathy was associated with an increased risk of AMD, possibly because the two diseases have common risk factors (e.g., age, obesity, and high dietary fat intake). Diabetes has been proposed to affect the development of AMD by altering hemodynamics and promoting the accumulation of advanced glycation end products [25]. Hyperglycemia also stimulates vascular endothelial growth factor (VEGF) [26, 27]. VEGF is a proinflammatory molecule contributing to blood-retinal barrier breakdown, which appears to be the key pathogenesis of diabetic retinopathy and AMD [25]. All of these theories support our finding that diabetic retinopathy is associated with AMD.

Our study revealed that metformin was associated with a lower risk of developing AMD. The underlying mechanism remains unclear but may be related to the antioxidative and anti-inflammatory properties of metformin. AMD involves a complex interplay of oxidative processes, inflammation, and immune reaction [28, 29]. Aging, light exposure, and lipid peroxidation produce excess amounts of reactive oxygen species (ROS), leading to oxidative stress and degeneration of the retinal pigment epithelium (RPE) [30]. RPE cell death activates an inflammatory reaction, which upregulates the expression of VEGF [31]. VEGF further stimulates ROS production [32], resulting in more oxidative stress and an inflammatory cascade. In previous studies, metformin decreased ROS levels, ameliorated oxidative stress [33–35], and inhibited inflammatory reactions [36, 37]. These effects may be associated with a lower risk of developing AMD. Similar protective effects of antioxidants (e.g., vitamin C, vitamin E, and zinc) against AMD were found in the Age-Related Eye Disease Study 1.

One strength of our study is that we investigated whether metformin has a dose-response risk-lowering effect for AMD, which further confirmed the association between metformin use and AMD. The lower adjusted HRs for AMD in those with longer durations of metformin treatment and higher metformin doses support the favorable effect of metformin. A study limitation is that the NHIRD does not include laboratory data, such as HbA1c or eGFR. However, we still found variables representing them. In our analyses, we considered the impact of diabetic retinopathy, which to some extent can be taken to represent the severity of diabetes. Besides, we adopted insulin treatment as a surrogate of poor diabetic control. And, the diagnosis of chronic kidney disease was used as a surrogate of impaired kidney function. Through the process of adjusting possible confounders and the PS-matching method, we have tried to reduce the confounding bias as possible as we could. It is another limitation that the NHIRD lacks data on lifestyle factors, such as smoking and dietary habits. More epidemiological studies or studies incorporating chart reviews are needed to collect such information.

Other strengths of our study are the large sample, long study period, and confirmed diagnoses with well-accepted ICD-9-CM codes. However, studies based on claims databases may have the limitation that the diagnostic codes in the database are not as accurate as those in clinical charts. Fortunately, in our healthcare system, the National Health Administration (NHA) frequently checks the cross-consistency of claims and chart data. The NHA also checks whether patients' diagnoses are confirmed through standard examination protocols. Therefore, the diagnoses in our database have a high degree of accuracy.

## 5. Conclusions

Using the LHID with a long study period of 13 years, we found that metformin was associated with a lower risk of AMD, and that the risk-lowering trend was significantly associated with a higher dose of metformin. These results were based on statistical analysis of data from a nationwide database. At present, we can only derive the association, not the causality. In addition, the underlying mechanism of the association remains to be explored. Further prospective studies and basic research will be undertaken to elucidate the possible explanations.

## Figures and Tables

**Table 1:** Characteristics of the study subjects.

Variable	Total (*n* = 68205)	Metformin group (*n* = 45524)	Nonmetformin group (*n* = 22681)	*p*-value
Age, years	56.1 ± 12.6	55.2 ± 12.6	57.8 ± 12.7	<0.0001
Age group, years				<0.0001
<50	22282 (32.7)	15640 (34.4)	6642 (29.3)	
50–60	20464 (30.0)	14393 (31.6)	6071 (26.8)	
60–70	14300 (21.0)	9501 (20.9)	4799 (21.1)	
≥70	11159 (16.3)	5990 (13.1)	5169 (22.8)	
Sex				<0.0001
Male	35892 (52.6)	24771 (54.4)	11121 (49.0)	
Female	32313 (47.4)	20753 (45.6)	11560 (51.0)	
Hypertension				<0.0001
Yes	46521 (68.2)	32004 (70.3)	14517 (64.0)	
No	21684 (31.8)	13520 (29.7)	8164 (36.0)	
Hyperlipidemia				<0.0001
Yes	45061 (66.1)	31575 (69.4)	13486 (59.5)	
No	23144 (33.9)	13949 (30.6)	9195 (40.5)	
Coronary artery disease				<0.0001
Yes	20838 (30.6)	14481 (31.8)	6357 (28.0)	
No	47367 (69.4)	31043 (68.2)	16324 (72.0)	
Obesity				
Yes	3505 (5.1)	2578 (5.7)	927 (4.1)	<0.0001
No	64700 (94.9)	42946 (94.3)	21754 (95.9)	
Diabetic retinopathy				<0.0001
Yes	5727 (8.4)	5294 (11.6)	433 (1.9)	
No	62478 (91.6)	40230 (88.4)	22248 (98.1)	
Chronic kidney disease				<0.0001
Yes	10843 (15.9)	5444 (12.0)	5399 (23.8)	
No	57362 (84.1)	40080 (88.0)	17282 (76.2)	
Medications				
Insulin	2886 (4.2)	1457 (3.2)	1429 (6.3)	<0.0001
Sulfonylurea	39624 (58.1)	25448 (55.9)	14176 (62.5)	<0.0001
DDP-4 inhibitor	10412 (15.3)	6738 (14.8)	3674 (16.2)	<0.0001
Thiazolidinedione	9256 (13.6)	6510 (14.3)	2746 (12.1)	<0.0001
Meglitinide	4324 (6.3)	2189 (4.8)	2135 (9.4)	<0.0001
*α*-glucosidase inhibitor	4090 (6.0)	2232 (4.9)	1858 (8.2)	<0.0001
Anti-hypertensives	40584 (59.5)	27953 (61.4)	12631 (55.7)	<0.0001
Lipid-lowering agents	39834 (58.4)	26994 (59.3)	12840 (56.6)	<0.0001
Follow-up period, years	6.7 ± 3.7	6.8 ± 3.7	6.7 ± 3.7	0.31
AMD during the follow-up period	2828 (4.2)	1563 (3.4)	1265 (5.6)	<0.0001

DPP-4: dipeptidyl peptidase-4; AMD: age-related macular degeneration. Data are presented as mean ± standard deviation or *n* (%).

**Table 2:** Risk factors for AMD in metformin users and nonusers.

Predictive variable	Univariate analysis		Multivariate analysis	
	Unadjusted HR (95% CI)	*p*-value	Adjusted HR (95% CI)	*p*-value
Metformin use (yes vs. no)	0.52 (0.48–0.57)	<0.0001	0.54 (0.50–0.58)	<0.0001
Age group, years				
<50	Reference		Reference	
50–60	2.63 (2.30–3.00)	<0.0001	2.57 (2.25–2.94)	<0.0001
60–70	4.79 (4.22–5.45)	<0.0001	4.54 (3.99–5.18)	<0.0001
≥70	7.24 (6.37–8.23)	<0.0001	6.44 (5.63–7.38)	<0.0001
Sex (male vs. female)	0.90 (0.83–0.97)	0.004	1.03 (0.96–1.11)	0.47
Hypertension	1.64 (1.52–1.77)	<0.0001	1.13 (1.04–1.22)	0.0003
Hyperlipidemia	1.11 (1.03–1.19)	0.006	1.10 (1.02–1.17)	0.008
Coronary artery disease	1.41 (1.31–1.52)	<0.0001	1.10 (1.01–1.20)	0.028
Obesity	1.49 (1.11–1.98)	<0.0001	1.28 (1.03–1.63)	0.030
Diabetic retinopathy	1.51 (1.37–1.67)	<0.0001	1.98 (1.78–2.20)	<0.0001
Chronic kidney disease	1.19 (1.08–1.31)	0.0004	1.05 (0.95–1.15)	0.36
Insulin treatment	1.16 (0.97–1.37)	0.10	1.04 (0.88–1.24)	0.63

AMD: age-related macular degeneration; HR: hazard ratio; CI: confidence interval. In the multivariate analysis, all variables in the table are included for adjustment.

**Table 3:** Characteristics of the PS-matched sample.

Variable	Metformin group (*n* = 22681)	Nonmetformin group (*n* = 22681)	*p*-value
Age, years	57.8 ± 12.6	57.8 ± 12.7	0.87
Age group, years			0.76
<50	6639 (29.3)	6642 (29.3)	
50–60	6088 (26.8)	6071 (26.8)	
60–70	4868 (21.5)	4799 (21.1)	
≥70	5086 (22.4)	5169 (22.8)	
Sex			0.91
Male	11136 (49.1)	11121 (49.0)	
Female	11545 (50.9)	11560 (51.0)	
Hypertension			0.82
Yes	14493 (63.9)	14517 (64.0)	
No	8188 (36.1)	8164 (36.0)	
Hyperlipidemia			0.70
Yes	31527 (59.6)	13486 (59.5)	
No	9154 (40.4)	9195 (40.5)	
Coronary artery disease			0.84
Yes	6378 (28.1)	6357 (28.0)	
No	16303 (71.9)	16324 (72.0)	
Obesity			
Yes	922 (4.1)	927 (4.1)	0.92
No	21759 (95.9)	21754 (95.9)	
Diabetic retinopathy			0.16
Yes	392 (1.7)	433 (1.9)	
No	22289 (98.3)	22248 (98.1)	
Chronic kidney disease			0.97
Yes	5403 (23.8)	5399 (23.8)	
No	17278 (76.2)	17282 (76.2)	
Medications			
Insulin	1409 (6.2)	1429 (6.3)	0.71
Sulfonylurea	14135 (62.3)	14176 (62.5)	0.70
DDP-4 inhibitor	3718 (16.4)	3674 (16.2)	0.59
Thiazolidinedione	2719 (12.0)	2746 (12.1)	0.71
Meglitinide	2109 (9.3)	2135 (9.4)	0.69
*α*-glucosidase inhibitor	1842 (8.1)	1858 (8.2)	0.80
Antihypertensives	12654 (55.8)	12631 (55.7)	0.84
Lipid-lowering agents	12831 (56.6)	12840 (56.6)	0.94
PS	0.60 ± 0.12	0.60 ± 0.13	0.80

PS: propensity score; DPP-4: dipeptidyl peptidase-4. Data are presented as mean ± standard deviation or *n* (%).

**Table 4:** Hazard ratios for AMD in PS-adjusted Cox regression.

Predictive variable	Multivariate analysis	
	Adjusted HR (95% CI)	*p*-value
Metformin use (yes vs. no)	0.57 (0.52–0.63)	<0.0001
PS	0.97 (0.89–1.16)	0.81

## Data Availability

Data are available from the National Health Insurance Research Database (NHIRD) published by Taiwan National Health Insurance (NHI) Bureau. The data utilized in this study cannot be made available in the manuscript, the supplemental files, or in a public repository due to the “Personal Information Protection Act” executed by Taiwan's government, starting from 2012. Requests for data can be sent as a formal proposal to the NHIRD (http://nhird.nhri.org.tw) or by e-mail to wt.gro.irhn@drihn.

## Abbreviations

AMD: Age-related macular degeneration
NHIRD: National Health Insurance Database
ICD-9-CM codes: International Classification of Diseases, Ninth Revision, Clinical Modification Codes
LHID: Longitudinal Health Insurance Database
eGFR: Estimated glomerular filtration rate
HbA1c: Hemoglobin A1c
HR: Hazard ratio
PS: Propensity score
CI: Confidence interval
VEGF: Vascular endothelial growth factor
ROS: Reactive oxygen species
RPE: Retinal pigment epithelium
NHA: National Health Administration.
